# Targeting SIRT-1/AMPK/Nrf2 Signaling Pathway by Tenofovir Protected Against Cyclophosphamide-Induced Nephrotoxicity and Cardiotoxicity in Rats

**DOI:** 10.3390/pharmaceutics17111467

**Published:** 2025-11-13

**Authors:** Yousef S. Alresheedi, Omnia A. Nour, Manar A. Nader, Marwa S. Zaghloul

**Affiliations:** 1 Department of Pharmacology and Toxicology, Faculty of Pharmacy, Mansoura University, Mansoura 35516, Egypt; yousef9291@gmail.com (Y.S.A.); asilsamy@gmail.com (O.A.N.); manarahna@mans.edu.eg (M.A.N.); 2 Department of Pharmacology and Toxicology, Faculty of Pharmacy, Mansoura National University, Gamasa 35761, Egypt

**Keywords:** cyclophosphamide, tenofovir, Nrf2/HO-1, SIRT1, Bcl-2, LC3

## Abstract

**Background/Objectives**: Cyclophosphamide (CYC) is a commonly used alkylating agent for treating various cancers and autoimmune disorders. However, its use is often hampered by serious side effects, affecting multiple organs. This study aimed to explore whether tenofovir (TFV), a nucleotide reverse transcriptase inhibitor, could offer protective benefits against CYC-induced organ toxicity in rats. **Methods**: Two different TFV doses (25 and 50 mg/kg) were tested. The researchers evaluated the effects of TFV on kidney and heart function biomarkers, oxidative stress, autophagy, apoptosis, and inflammatory markers. **Results**: The results showed that pre-treatment with TFV significantly reduced the harmful effects of CYC, as evidenced by decreasing the activity of serum lactate dehydrogenase (LDH) and creatine kinase-myocardial band (CK-MB), and the levels of serum creatinine (Cr.), blood urea nitrogen (BUN), and malondialdehyde (MDA). TFV also boosted antioxidant defenses by increasing the expression of key proteins such as Nrf2/HO-1, AMPK, and SIRT1. Also, TFV regulated inflammatory and apoptotic pathways (revealed by reducing IL-1β level and increasing Bcl-2 level) and improved autophagy (showed by reducing LC3 expression). **Conclusions**: Overall, these findings suggested that TFV has strong protective effects against CYC-induced organ toxicity, likely through its anti-inflammatory, antioxidant, and anti-apoptotic mechanisms. This points to TFV as a potential therapeutic agent to help mitigate the organ damage caused by CYC.

## 1. Introduction

With an emphasis on the tumor cells’ ability to proliferate and spread, chemotherapeutic drugs have been used to treat cancer for approximately 70 years. Strong chemotherapeutic drugs have been developed, but the main obstacles to their successful clinical use remain to be their toxicity to healthy tissues, adverse side effects across a range of organ systems, and drug resistance [1]. Cyclophosphamide (CYC) is one of the chemotherapeutic medicines that suppresses the immune system and fights cancerous cells.

Cyclophosphamide (CYC) belongs to the alkylating agent class of cancer medications that prevent cancer cells from growing and lead to the elimination of cancer cells. However, it can also impact healthy body cells, which could result in dangerous side effects [2]. It is used to treat various neoplastic illnesses including those of the ovary, breast, blood, lymphatic system, retinoblastoma, multiple myeloma, and mycosis fungoides as well as autoimmune disorders [3,4]. The guanine base of DNA gains an alkyl group thanks to the alkylating properties of CYC. This results in the synthesis of aberrant cytosine–thymine pairs, which drives the cells’ DNA repair system to eliminate the altered guanine and ultimately results in cell death [5]. CYC is documented to have the ability to induce the damage of liver, kidneys, heart, lung, testicular tissues, and urinary blader in a dose dependent manner [6,7,8,9,10].

CYC-induced organ toxicity is predominantly driven by excessive inflammation and oxidative stress, a conclusion now underscored by recent experimental studies and mechanistic reviews. When CYC undergoes metabolic activation in the body, it leads to the generation of reactive oxygen species (ROS) and depletion of cellular antioxidant defenses such as glutathione (GSH), superoxide dismutase, and catalase [11,12]. This oxidative imbalance results in widespread lipid peroxidation, DNA damage, and protein carbonylation, thus impairing cellular integrity in the affected areas [13,14]. Simultaneously, CYC robustly triggers the expression of pro-inflammatory mediators—most notably through upregulation of the nuclear factor kappa B (NF-κB) pathway and subsequent release of cytokines such as tumor necrosis factor-alpha (TNF-α) and Interleukin-6 (IL-6)—fueling a cascade of tissue inflammation, edema, and fibrotic changes [15]. These intertwined mechanisms—massive oxidative insult paired with inflammatory signaling—are now recognized as the molecular underpinning for the multi-organ toxicity observed during CYC therapy. Given these limitations of CYC, there is increasing interest in repurposing drugs with anti-inflammatory and cytoprotective properties, such as tenofovir (TFV) to be used in the attenuation of CYC-induced organ toxicity.

Tenofovir (TFV) is a nucleotide analog with antiviral activity against retroviruses such as human immunodeficiency virus (HIV-1), chronic hepatitis B virus (HBV) infections, and hepadnaviral infection. Following its administration, it is rapidly converted to its active form, tenofovir diphosphate. Tenofovir has a longer half-life than other nucleoside analogs, supporting once-daily dosing. It is not metabolized by human cytochrome P450 enzymes and there are few drug interactions [16].

In addition to its antiviral activity, anti-inflammatory activity of TFV has also been reported. TFV can reduce inflammation by inhibiting the release of inflammatory messengers such as Interleukin-8 (IL-8) and maintaining a stable balance of interleukins such as IL-8 and IL-10. Its anti-inflammatory effect helps boost the immune system of individuals with HIV, making them less susceptible to other infections [17]. Furthermore, Calza et al. (2016) demonstrated that TFV–emtricitabine dramatically decreased endothelial activation and serum markers of inflammation such as IL-6, TNF-α, intercellular adhesion molecule-1 (ICAM-1), vascular cell adhesion protein-1 (VCAM-1), Eselectin, and P-selectin in HIV-infected antiretroviral naive patients [18]. Additionally, Dysangco et al. (2017) imply that TFV may be most suited for people living with HIV, in order to lower the risk of cardiovascular disease linked to endothelial cells via enhanced ectonucleotides activities [19]. In addition, a pre-clinical model employing a tenofovir–silver nanoparticle conjugate in diabetic rats observed increased glutathione, SOD, and CAT (antioxidant enzymes), reduced malondialdehyde and IL-1β (oxidative/inflammatory markers), and improved cognitive outcomes [20].

Based on these observations, we hypothesized that TFV could attenuate CYC-induced renal and cardiac toxicity. Thus, we investigated its possible protective effects at two distinct dosages and the underlying mechanisms in a rat model.

## 2. Materials and Methods

### 2.1. Animals

Forty adult male Wistar rats, weighing 250 ± 20 g at fifteen weeks of age, were purchased from Medical Experimental Research Center (MERC), Faculty of Medicine, Mansoura University, Mansoura, Egypt. The rats were housed in plastic polypropylene cages with wood chips for bedding (eight rats/cage). All animal experiments have been performed in accordance with the Basic & Clinical Pharmacology & Toxicology (BCPT) policy for experimental and clinical studies [21] and were accepted by the Faculty of Pharmacy’s Institutional Animal Care and Use Committee (ACUC) at Mansoura University in Egypt (Approval no: PHARM.PhD.23.1.15). All the rats were allowed seven days to get used to the experimental environment before the experiment started. Animals had ad libitum access to water and standard laboratory chow, under controlled temperature (22–25 °C) and a 12 h light/dark cycle.

Animals’ welfare was closely monitored daily throughout the experiment to avoid, minimize and alleviate any sort of pain and distress via daily observing the rats for any changes that could reflect pain or distress. Welfare assessments included monitoring of any changes in general appearance (e.g., posture and grooming), behavior (e.g., activity and vocalization), body weight and food/water intake, and also clinical signs (e.g., respiration rate and mobility). Monitoring was performed by trained staff familiar with the species’ normal behavior and appearance. Animals excluded from data analysis included animals suffering from signs of unexpected illness and infection in addition to animals that failed to receive the full dose of CYC or TFV owing to technical errors in drug administration or any problems during anesthesia or sample collection.

### 2.2. Drugs and Chemicals Utilized

Cyclophosphamide (CYC) was obtained as Endoxan^®^ (1 g vial, Baxter Oncology GmbH, Kantstraße 2, 33790 Halle/Westfalen, Germany). The product is lyophilized powder intended for intravenous use. Stored at ≤25 °C before reconstitution; after dissolution, solution was kept at 2–8 °C until administration. Tenofovir (TFV) was purchased as tenofovir disproxil fumarate powder from sigma-Aldrich (Saint Louis, MO, USA). It was suspended in carboxymethyl cellulose (CMC) (0.5% *w*/*v*) to prepare a uniform suspension just before being given orally. All other chemicals were of analytical grade.

### 2.3. Experimental Design

**Figure 1:**
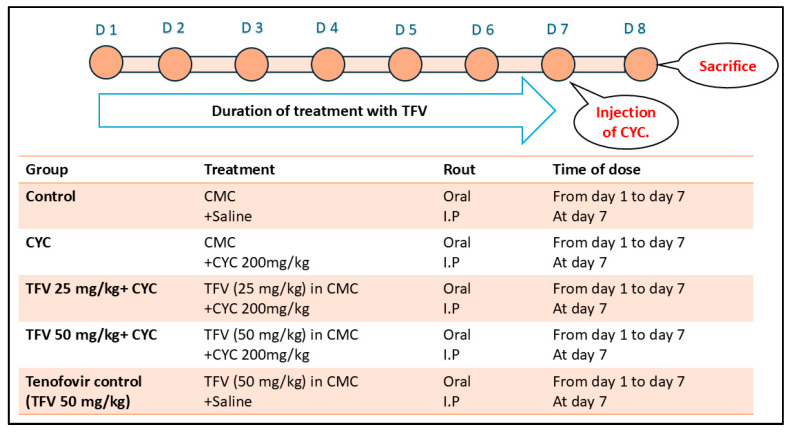
**Experimental Design**: Rats were randomly split up into five groups (n = 8) (i) The control group. (ii) cyclophosphamide (CYC) group. (iii) tenofovir 25 mg/kg group (TFV 25 mg/kg + CYC). (iv) tenofovir 50 mg/kg group (TFV 50 mg/kg + CYC). (v) tenofovir control (TFV 50 mg/kg) group. D: day, CMC: carboxymethyl cellulose, CYC: cyclophosphamide, I.P: intraperitoneal; TFV: tenofovir.

Forty male Wistar rats were randomly split up into five groups, each with eight rats. (i) The control group: rats received regular oral CMC for 7 days followed by single intraperitoneal (i.p) injection of normal saline at the 7th day of the study. (ii) cyclophosphamide (CYC) group: rats received regular oral CMC for seven days followed by single i.p injection of CYC at a dose of 200 mg/kg on the seventh day of the study [22]. (iii) tenofovir 25 mg/kg group (TFV 25 mg/kg+ CYC): rats were given a low dose of TFV (25 mg/kg/day) suspended in CMC orally via gavage from day one to day seven of the study, and on day seven, they were given a single i.p injection of CYC [23]. (iv) tenofovir 50 mg/kg group (TFV 50 mg/kg+ CYC): during the first seven days, rats were given an oral gavage of a high dose of TFV (50 mg/kg/day) at the same time each day [23] and a single i.p injection of CYC on the 7th day. (v) tenofovir control (TFV 50 mg/kg) group: rats were given an oral gavage of a high dose of TFV (50 mg/kg/day) only for 7 days. The rats were sacrificed 24 h after receiving the injection of CYC or its vehicle (Figure 1). We followed a 2-step euthanasia process; first, rats were anesthetized with thiopental sodium (40 mg/kg, i.p.) for blood collection followed by exsanguination via cardiac puncture.

### 2.4. Samples Collection

#### 2.4.1. Urine Gathering

On the 7th day, after the injection of CYC or saline, metabolic cages were used to gather urine samples. The cages feature a wire-grid floor, a lower collection chamber with a special funnel that separates excrement and urine that falls through the grid floor into two categories, and a circular upper section housing the rat. Animals were instantly kept individually in metabolic cages to collect urine for 24 h. As animals were kept individually, each animal’s urine was collected in a separate, clearly labeled tube and stored at −20 °C. The rats were provided with constant volume of water and deprived of food throughout 24 h.

Rats were closely monitored for any signs of stress or discomfort during confinement in metabolic cages including changes in activity level, respiration rate, and vocalization. If any animal exhibited persistent distress (e.g., agitation, vocalization, or self-injury), confinement was immediately terminated, and the animal was allowed to recover in its home cage. Monitoring was performed by trained personnel at 2 h intervals.

#### 2.4.2. Blood and Tissue Collection

Twenty-four hours after CYC injection, the animals were weighed and venous blood samples were obtained from the retro-orbital plexus. The blood was centrifuged at 5000 rpm for 15 min, and the serum was stored at −80 °C until it was needed for assessments. It took little time to remove and weigh the kidney and heart. Tissue slices from each organ were removed, paraffin embedded, and then stored in 10% formalin saline for use in immunohistochemical and pathological examinations. The remaining organ tissues were homogenized using an Omni tissue homogenizer (Omni International, Inc., Kennesaw, GA, USA) in 20% *w*/*v* ice-cold phosphate buffer (0.01 M, pH 7.4). The homogenate of different organ tissues was centrifuged at 3000 rpm at 4 °C for 20 min using a cooling centrifuge (Sigma 3K30 HS Refrigerated and heated benchtop centrifuge); aliquots were made from the acquired supernatant and stored at −80 °C till use.

### 2.5. Assessments

#### 2.5.1. Assessment of Serum Lactate Dehydrogenase (LDH), Creatine Kinase-Myocardial Band (CK-MB), Creatinine (Cr), and Blood Urea Nitrogen (BUN) Altered via CYC Injection

Serum LDH (for indication of multiple organ damage), CK-MB (for indication of cardiac injury), and serum Cr and BUN (for indication of renal injury) were evaluated using commercially available kits: LDH (SWEMED diagnostic, Karnataka, India), CK-MB, and Cr and BUN (SPINREACT, Carretera Santa Coloma, Girona, Spain), respectively. The absorbance was read using a spectrophotometer (Labomed, Los Angeles, CA, USA). The assessment procedures were performed according to the manufacturer guidelines in duplicate manner.

#### 2.5.2. Assessment of Urine Total Protein (TP) and Creatinine (Cr) Altered via CYC Injection

TP and Cr in urine were quantified using commercially available kits, Bio-Diagnostic (Giza, Egypt), and SPINREACT (Carretera Santa Coloma, Girona, Spain), respectively, according to manufacturer protocol in duplicate manner.

#### 2.5.3. Assessment of Renal and Cardiac Malondialdehyde (MDA) Content and Total Antioxidant Capacity (TAC)

Determination of MDA and TAC levels was carried out using commercially available kits: MDA (My BioSource, San Diego, CA, USA, cat. No; MBS268427) and TAC (Cell Biolabs Inc., San Diego, CA, USA, cat. No; STA-360) according to manufacturer protocol in duplicate manner.

#### 2.5.4. Enzyme-Linked Immunosorbent Assay (ELISA) of Renal and Cardiac AMP-Activated Protein Kinase (AMPK), Nuclear Factor Erythroid 2-Related Factor 2 (Nrf2), Heme Oxygenase-1 (Ho-1), B-Cell Lymphoma-2 (Bcl-2), and Silent Information Regulator Sirtuin 1 (Sirt1) Levels

Double-antibody sandwich ELISA kits were utilized following the producers’ commands in duplicate manner: AMPK (R&D system, Minneapolis, MN, USA, # DYC3197), Nrf2 (BT LAB, Beijing, China, #E1083Ra), HO-1 (CUSABIO, Houston, TX, USA #CSB-E08267r), Bcl-2 (CUSABIO, Houston, TX, USA, #CSB-E08854r), and SIRT1 (BT LAB Beijing, China, #E1145Ra). ELISA assays were performed using tissue homogenate and the concentration was expressed as ng/mg tissue. The used microplate reader (Bio Tek instruments ELx800, Winooski, VT, USA) was set to λ = 450 nm.

### 2.6. Histopathological Examination of Renal and Cardiac Tissues

The kidney and heart were dissected from rats and fixed in neutral-buffered formalin for 24 h. The samples were dehydrated in ascending grades of alcohol, cleared with xylene, and embedded in paraffin wax. The embedded samples were cut with microtome at 5 μm thick sections. The sections were stained with hematoxylin and eosin (H&E) and then examined blindly under the light microscope (Olympus CH2, Tokyo, Japan) and the slides were coded prior to examination. Examination was recorded in 4 sections per slide (each slide represents one animal) and the average of each 4 scores was calculated and used for statistical analysis and scattered dot graph presentation. To quantify tissue damage, a semi-quantitative scoring system was applied based on established methods [24].

### 2.7. Immunohistochemical Assessment of Renal and Cardiac Expression of Interleukin-1B (IL-1B) and Microtubule-Associated Protein 1A/1B-Light Chain 3 (LC3)

The expression levels of renal and cardiac IL-1B (Cat. No. GB11113, Servicebio, East Lake High-Tech Development Zone, Wuhan, China) and LC3 (Cat. No. A19665, ABclonal, Woburn, MA, USA) were assessed by immunostaining using the Avidin-Biotin Complex method [25]. The used antibody dilutions were 1:1000 and 1:100 for IL-1B and LC3, respectively. The stained slides were examined blindly under the light microscope (Olympus CH2, Japan) and the slides were coded prior to examination. Examination was recorded in 4 sections per slide (each slide represents one animal), one photo was taken per section, and the average of each 4 results was calculated and used for statistical analysis. The whole tissue sections with high-resolution images were entered into image j software. A virtual dissection or extracting the ROI (region of interest) in whole tissue sections at magnifications up to 40× was performed to estimate relative antigen content. The digital image is processed using the color deconvolution method to separate the brown DAB chromogen from the hematoxylin counterstain on a microscope slide. A monochrome image representing the DAB content is then subjected to frequency analysis using ImageJ Software (FIJI 2.10.0, National Institutes of Health, MD, USA) and calculation of the area fraction of DAB (antigen) was conducted. The result is expressed as a percentage of the immunopositively stained area in each renal and cardiac sections and expressed as means ± SEM [26].

### 2.8. Statistical Analysis

The statistical analysis was conducted by GraphPad Prism 8.0.1(GrapPad Prism Inc., San Diego, CA, USA) software to evaluate the differences between the experimental groups utilizing one-way analysis of variance (ANOVA) after confirming the assumptions of normality and homogeneity of variance. Post hoc test and Tukey–Kramer test were conducted to identify specific pairwise differences, indicating significant comparisons among groups. In cases where data did not meet the generality or symmetric perception of the variance, non-parametric tests were used. Kruskal–Wallis’s test was used to compare the difference between three independent groups. If significant differences (*p* < 0.05) were detected, Dunn’s post hoc test was used for comparison and controlled several tests. Descriptive statistics, presented as means ± standard error (SEM) for parametric data and median and interquartile range for non-parametric ones, were used to summarize the data, highlighting the central tendency and variability within each group (*p* < 0.005).

## 3. Results

### 3.1. The Impact of TFV (TFV 25 and 50 mg/kg) on Serum LDH, CK-MB, Cr, and BUN Altered via CYC Injection

**Figure 2:**
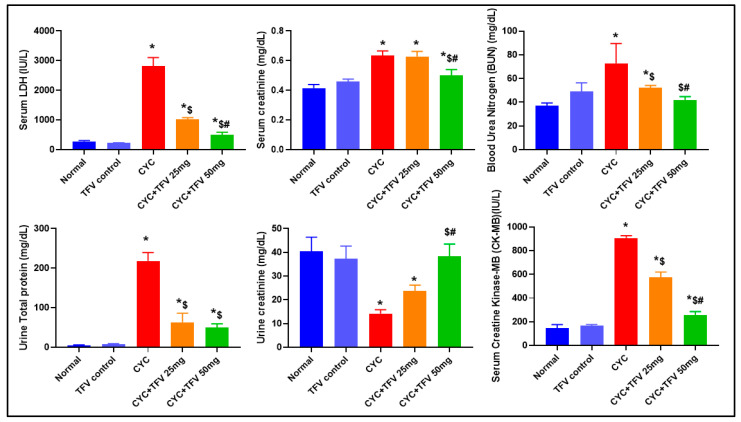
**The impact of tenofovir (TFV) on serum lactate dehydrogenase (LDH), creatine kinase-myocardial band (CK-MB), serum creatinine (Cr), blood urea nitrogen (BUN), urine total protein (TP), and urine Cr altered via cyclophosphamide (CYC) injection.** Data are expressed as mean ± standard error of the mean (SEM); n = 8. CYC (200 mg/kg, single i.p dose); TFV (25 or 50 mg/kg, oral for 7 days). The values of *, ^$^, and ^#^ are significantly different from those of the control, CYC, and the (CYC + TFV 25 mg/kg) groups, respectively. This was determined using one-way ANOVA followed by Tukey–Kramer multiple comparison post-hoc tests (*p* < 0.05).

As shown in Figure 2, the injection of CYC into rats resulted in a significant elevation in serum LDH, CK-MB, Cr, and BUN levels by about 10.12, 6.12, 1.53, and 1.97 folds, respectively, in contrast to the normal group. On the other hand, the pre-treatment with TFV disclosed a significant reduction in these markers compared to the CYC group. Interestingly, TFV (25 mg/kg) revealed a significant decrease in serum LDH, CK-MB, and BUN levels only by approximately 63.8%, 36.6%, and 28.2%, respectively, in contrast to the CYC group. However, TFV (50 mg/kg) disclosed a significant decrease in serum LDH, CK-MB, Cr, and BUN levels by approximately 82.1%, 71.5%, 21.5%, and 42.3%, respectively, in contrast to the CYC group. Additionally, there was a significant decrease in serum LDH, CK-MB, and Cr levels in the TFV (50 mg/kg) group in comparison to the TFV (25 mg/kg) group by about 50.6%, 54.9%, and 20.5%, respectively.

### 3.2. The Impact of TFV (TFV 25 and 50 mg/kg) on Urine TP and Cr Altered via CYC Injection

As illustrated in Figure 2, injection of CYC significantly elevated urine TP levels by about 44 folds in contrast to normal, indicating kidney damage. Furthermore, CYC + TFV 25 mg/kg and CYC + TFV50 mg/kg groups significantly reduced urine TP levels compared to the CYC group by approximately 71% and 77%, respectively. Moreover, CYC injection resulted in a significant decrease in urine creatinine level by about 65% compared to the control group, which means a reduction in glomerular filtration rate (GFR) and renal damage in contrast to normal rats. On the other hand, the treatment with TFV 25 mg/kg and 50 mg/kg resulted in elevation in urine creatinine by approximately 1.7 and 2.7 folds, respectively, in contrast to the CYC group. Interestingly, there was a significant elevation in urine creatinine levels in the CYC + TFV 50 mg/kg group compared to the CYC + TFV 25 mg/kg group by about 1.6 folds.

### 3.3. The Impact of TFV (TFV 25 and 50 mg/kg) on Renal and Cardiac Histopathology Alteration Caused by CYC

#### 3.3.1. Renal Tissue

**Figure 3:**
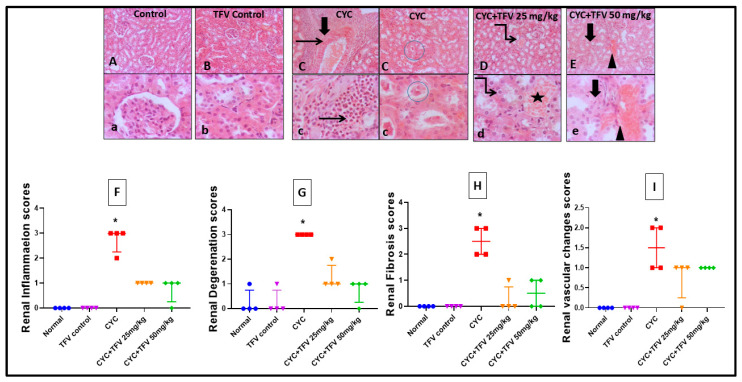
**The impact of tenofovir (TFV) on renal histopathological alteration and damage scoring induced by cyclophosphamide (CYC) using hematoxylin and eosin stain.** (**A**,**a**) Control group, (**B**,**b**) TFV control group, (**C**,**c**) CYC group, (**D**,**d**) CYC + TFV 25 group, (**E**,**e**) CYC + TFV 50 group. (**F**) Scatter dot plots displaying renal inflammation scores, (**G**) scatter dot plots displaying renal degeneration scores, (**H**) scatter dot plots displaying renal fibrosis scores, (**I**) scatter dot plots displaying renal vascular changes scores. Thin arrow: inflammation; thick arrow: interstitial fibrosis; arrowhead: intestinal congestion; twisted arrow: tubular degeneration or necrosis; circle: intraluminal cast; star: hemorrhage. Upper raw original magnification 10×, lower raw 40×, and scale bar 100 µm and 20 µm, respectively. CYC (200 mg/kg, single i.p dose); TFV (25 or 50 mg/kg, oral for 7 days). Data are presented as median ± IQR range; n = 4. The values of * were significantly different from those of the control group, as found with a Kruskal–Wallis test followed by a Dunn multiple comparison test (*p* ˂ 0.05).

Regarding renal tissues, as illustrated in Figure 3, the injection of CYC into rats resulted in significant diffuse tubular degeneration with severe tubular epithelial vacuolation, with extensive perivascular fibrosis admixed with marked numbers of eosinophils and neutrophils replacing most of the renal parenchyma with diffuse intraluminal hyaline cast when compared to the control group. On the other hand, the TFV 25 mg/kg + CYC group showed mild tubular degeneration with mild interstitial congestion, while the TFV 50 mg/kg + CYC group showed mild to moderate interstitial congestion with mild interstitial fibrosis when compared to the CYC group.

Additionally, renal histopathological scoring showed that CYC injection induced significant elevation in renal inflammation, degeneration, fibrosis, and renal vascular change scores, while co-treatment with TFV 25 and 50 mg/kg markedly reduced all these histopathological scores, indicating the protective effect of TFV against CYC-induced renal damage.

#### 3.3.2. Cardiac Tissue

**Figure 4:**
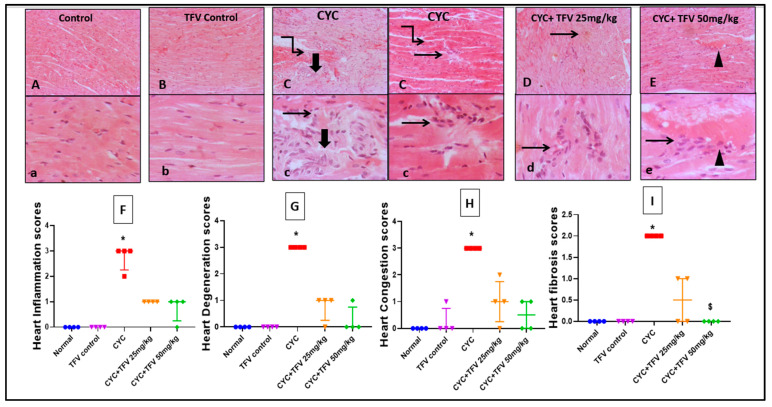
**The impact of tenofovir (TFV) on cardiac histopathological alteration and damage scoring induced by cyclophosphamide (CYC) using hematoxylin and eosin stain.** (**A**,**a**) Control group, (**B**,**b**) TFV control group, (**C**,**c**) CYC group, (**D**,**d**) CYC + TFV 25 group, (**E**,**e**) CYC + TFV 50 group. (**F**) Scatter dot plots displaying cardiac inflammation scores, (**G**) scatter dot plots displaying cardiac degeneration scores, (**H**) scatter dot plots displaying cardiac congestion scores, (**I**) scatter dot plots displaying cardiac fibrosis scores. Twisted arrow: myocardial necrosis; thick arrow: fibrosis; thin arrow: inflammation; arrowhead: vascular congestion. Upper raw original magnification 10×, lower raw 40×, and scale bar 100 µm and 20 µm, respectively. CYC (200 mg/kg, single i.p dose); TFV (25 or 50 mg/kg, oral for 7 days). Data are presented as median ± IQR range; n = 4. Values of * and ^$^ were significantly different from those of the control and CYC groups respectively, as found with the Kruskal–Wallis test followed by a Dunn multiple comparison test (*p* ˂ 0.05).

Regarding cardiac tissues, as illustrated in Figure 4, the injection of CYC into rats resulted in sever myocardial necrosis with shrunken, hyper eosinophilic cytoplasm with faded nuclei surrounded either by focal inflammatory cells or coalescing fibrosis mixed with abundant macrophages, lymphocytes, and plasma cells when compared to the control group. On the other hand, TFV 25 mg/kg + CYC showed few scattered inflammatory cells, while TFV 50 mg/kg + CYC showed mildly congested blood vessels with few to mildly inflammatory perivascular cells including macrophages, lymphocytes, and fibroblasts when compared to the CYC group.

Moreover, cardiac histopathological scoring showed that CYC injection induced significant increase in cardiac inflammation, degeneration, congestion, and fibrosis scores, while co-treatment with TFV 25 and 50 mg/kg markedly reduced histopathological damage scores in cardiac tissues, indicating the protective effect of TFV against CYC-induced cardiac damage.

After the initial assessment and histopathological examination which proved that a high dose (50 mg/kg) of TFV is more effective than a low dose (25 mg/kg) in improving tissue damage caused by CYC, and for ease of data presentation, the rest of this study has been carried out on the high dose group for examination of the underlying molecular mechanisms.

### 3.4. The Impact of TFV (50 mg/kg) on Renal and Cardiac MDA and TAC Altered via CYC Injection

**Figure 5:**
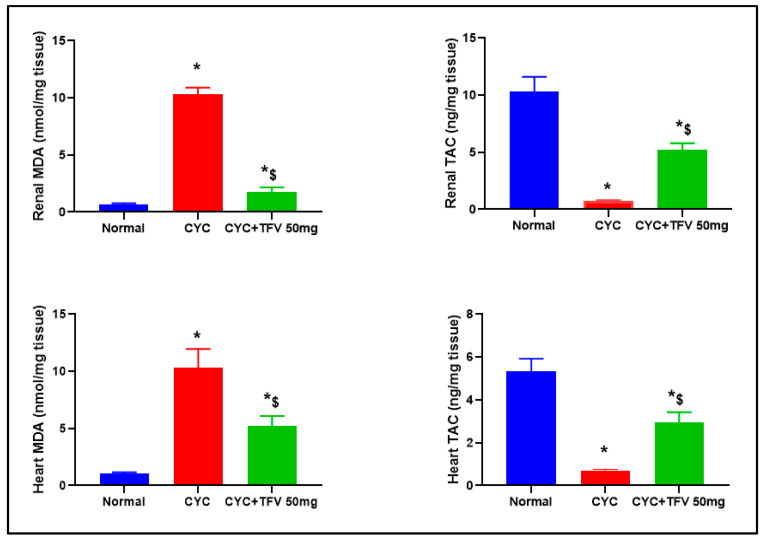
**The impact of tenofovir (TFV) on renal and cardiac malondialdehyde (MDA) levels and total antioxidant capacity (TAC) altered via cyclophosphamide (CYC) injection.** Data are expressed as mean ± standard error of the mean (SEM); n = 6. CYC (200 mg/kg, single i.p dose); TFV (50 mg/kg, oral for 7 days). The values of * and ^$^ are significantly different from those of the control and CYC groups, respectively. This was determined using one-way ANOVA followed by Tukey–Kramer multiple comparison post hoc tests (*p* < 0.05).

As illustrated in Figure 5, the injection of CYC into rats resulted in significant elevation of renal and cardiac MDA, by 15.96 and 9.78 folds, respectively, parallel with significant reduction in renal and cardiac TAC levels by 13.6 and 7.86 folds, respectively, in contrast to the normal group. On the other hand, treatment with TFV 50 mg/kg resulted in significant decrease in renal and cardiac MDA level, by 80.62% and 49.02%, respectively, in contrast to the CYC group.

### 3.5. The Impact of TFV (50 mg/kg) on Renal and Cardiac AMPK, Nrf2, HO-1, Bcl-2, and SIRT1 Altered via CYC Injection

**Figure 6:**
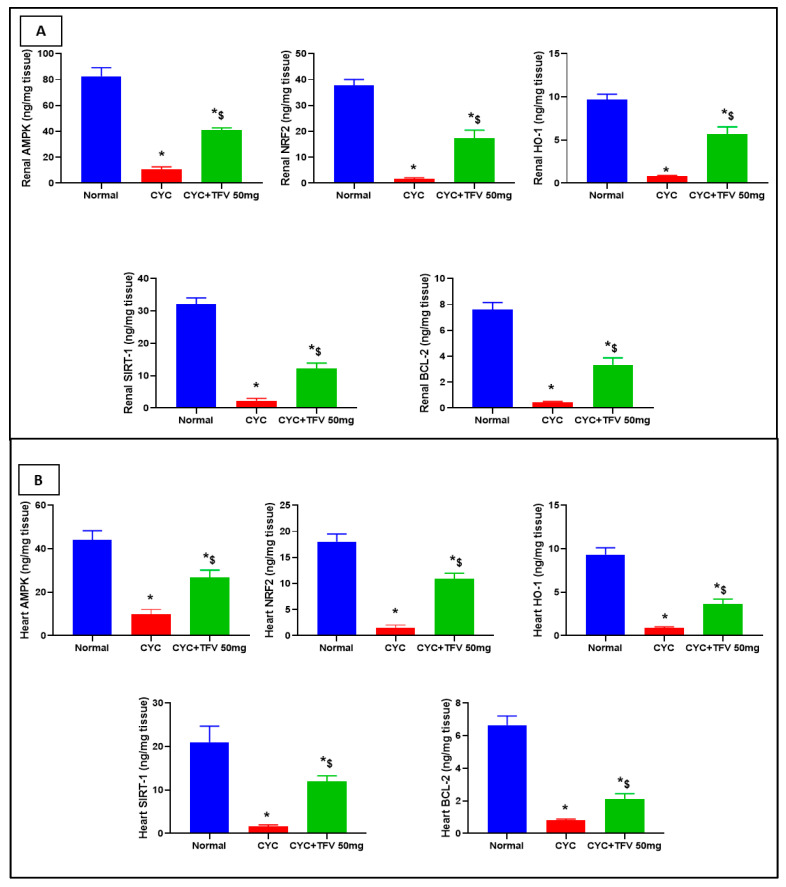
**The impact of tenofovir (TFV) on renal and cardiac AMP-activated protein kinase (AMPK), nuclear factor erythroid 2-related factor 2 (Nrf2), Heme oxygenase-1 (HO-1), B-cell lymphoma-2 (Bcl-2), and silent information regulator Sirtuin 1 (SIRT1) altered via cyclophosphamide (CYC) injection.** (**A**): Protein levels in renal tissues. (**B**): Protein levels in cardiac tissues. Data are expressed as mean ± standard error of the mean (SEM); n = 6. CYC (200 mg/kg, single i.p dose); TFV (50 mg/kg, oral for 7 days). The values of * and ^$^ are significantly different from those of the control and CYC groups, respectively. This was determined using one-way ANOVA followed by Tukey–Kramer multiple comparison post hoc tests (*p* < 0.05).

As illustrated in Figure 6A,B, the injection of CYC into rats resulted in significant downregulation in renal levels of AMPK, Nrf2, HO-1, Bcl-2, and SIRT1 by 7.52, 23.39, 12.2, 16.44, and 14.32 folds, respectively, compared to the control group, parallel with significant reduction in cardiac levels of AMPK, Nrf2, HO-1, Bcl-2, and SIRT1 by 4.41, 8.2, 10.43, 11.69, and 13.19 folds, respectively, in contrast to the normal group.

On the other hand, the treatment with TFV 50 mg/kg resulted in a significant elevation of renal Nrf2, HO-1, AMPK, Bcl-2, and SIRT1 by 10.75, 7.15, 3.72, 7.22, and 5.47, respectively, in contrast to the CYC group. Additionally, TFV 50 mg/kg resulted in a significant elevation in cardiac Nrf2, HO-1, AMPK, Bcl-2, and SIRT1 by 7.05, 4.11, 2.68, 2.63, and 7.55 folds, respectively, in contrast to the CYC group.

### 3.6. The Impact of TFV (50 mg/kg) on Renal and Cardiac IL-1B and LC3 Alteration Induced by CYC and Immunohistochemically Analyzed

**Figure 7:**
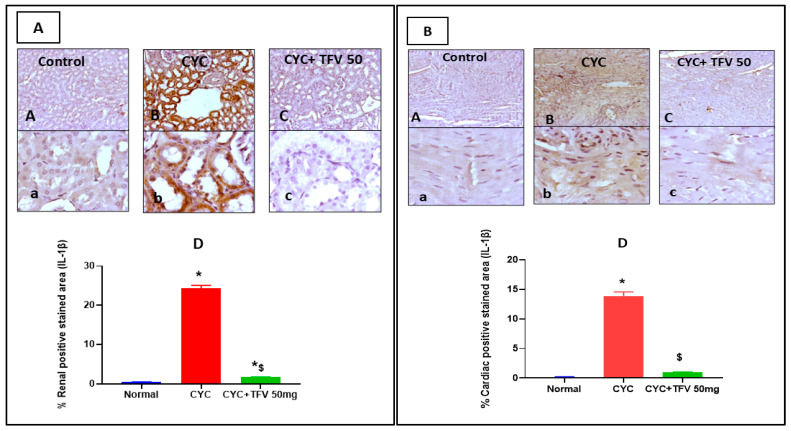
**The impact of tenofovir (TFV) on renal and cardiac expression level of Interleukin-1B (IL-1B) and microtubule-associated protein 1A/1B-light chain 3 (LC3) altered via cyclophosphamide (CYC) injection.** (**A**): IL-1B expression level in renal tissues. (**B**): IL-1B expression level in cardiac tissues. (**A**,**a**) Control group, (**B**,**b**) CYC group, (**C**,**c**) CYC + TFV 50 group, (**D**) column bar graph for the percentage of positive-stained area. Upper raw original magnification 10×, lower raw 40×, and scale bar 100 µm and 20 µm, respectively. Data are expressed as mean ± standard error of the mean (SEM); n = 4. CYC (200 mg/kg, single i.p dose); TFV (50 mg/kg, oral for 7 days). The values of * and ^$^ are significantly different from those of the control and CYC groups, respectively. This was determined using one-way ANOVA followed by Tukey–Kramer multiple comparison post hoc tests (*p* < 0.05).

As presented in Figure 7A, the CYC group revealed diffuse intense IL-1B immunopositively staining in cytoplasm of renal tubular epithelial cells compared to the control group, while the CYC + TFV 50 group showed few faint immunopositively stained tubular epithelial cells when compared to the CYC group. Additionally, in cardiac tissues, the CYC group showed diffuse sarcoplasmic IL-1B immunostaining myocardial fibers and more invading inflammatory cells compared to the control group, while the CYC + TFV 50 group showed a faint sarcoplasmic expression of IL-1b in myocytes compared to the CYC group (Figure 7B).

On the other hand, image analysis revealed that the percentage of positively stained area against IL-1B in renal and cardiac tissues was significantly elevated in the CYC group by about 48 and 71 folds, respectively, compared to the control group. Meanwhile, co-treatment with TFV 50 mg/kg significantly reduced the percentage of positively stained area in renal and cardiac tissues by about 92% and 93%, respectively, compared to the CYC group (Figure 7A,B).

**Figure 8:**
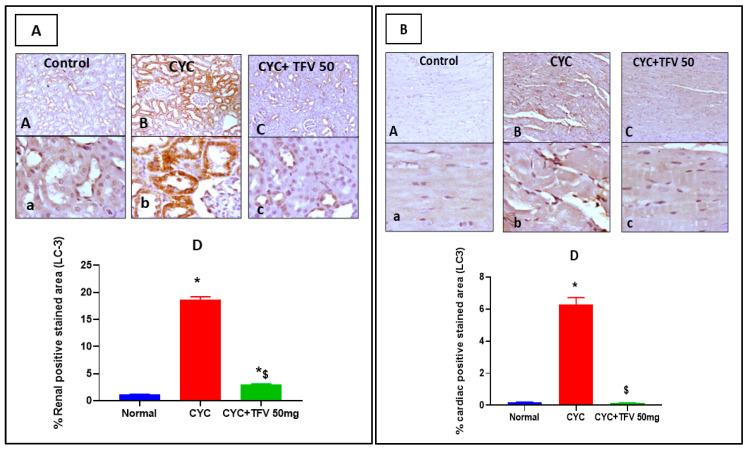
**The impact of tenofovir (TFV) on renal and cardiac expression level of microtubule-associated protein 1A/1B-light chain 3 (LC3) altered via cyclophosphamide (CYC) injection.** (**A**): LC3 expression level in renal tissues. (**B**): LC3 expression level in cardiac tissues. (**A**,**a**) Control group, (**B**,**b**) CYC group, (**C**,**c**) CYC + TFV 50 group, (**D**) column bar graph for percentage of positively stained area. Upper raw original magnification 10×, lower raw 40×, and scale bar 100 µm and 20 µm, respectively. Data are expressed as mean ± standard error of the mean (SEM); n = 4. CYC (200 mg/kg, single i.p dose); TFV (50 mg/kg, oral for 7 days). The values of * and ^$^ are significantly different from those of the control and CYC groups, respectively. This was determined using one-way ANOVA followed by Tukey–Kramer multiple comparison post hoc tests (*p* < 0.05).

Regarding LC3 expressions levels, as demonstrated in Figure 8A,B, the CYC group showed significant upregulation of cytoplasmic expression of LC3 in renal tubules compared to the control group, while the CYC + TFV 50 group showed focal coalescing mild to moderate faint cytoplasmic expression of LC3 in renal tubules when compared to the CYC group. Moreover, in cardiac tissues, the CYC group revealed significant immunopositively stained cytoplasmic myocytes with few positive inflammatory cells compared to the control group, while the CYC + TFV 50 group showed few faintly stained cytoplasmic myocytes when compared to the CYC group.

This was in line with the results of image analysis that revealed that the percentage of positively stained area against LC3 in renal and cardiac tissues was significantly elevated in the CYC group by about 16 and 34 folds, respectively, compared to the control group, while co-treatment with TFV 50 mg/kg significantly reduced the percentage of positively stained area in renal and cardiac tissues by about 84% and 97%, respectively, compared to the CYC group (Figure 8A,B).

## 4. Discussion

The results of our study revealed that the administration of TVF (25 and 50 mg/kg) improved the renal and cardiac injury induced by CYC (200 mg/kg) injection. The injection of CYC disclosed a significant elevation in serum LDH, Cr, BUN, and urine TP concomitant with the destruction of normal architecture of renal and cardiac tissues indicated through histopathological examination. This is consistent with findings from previous studies in which CYC has been associated with dose-dependent toxicity in vital organs [27,28,29]. On the other hand, pre-treatment of rats with TFV significantly attenuated these elevations, particularly at the higher dose (50 mg/kg), which led to decreases in LDH, creatinine, BUN, and urine TP.

CYC is metabolized into acrolein and phosphoramide mustard which produce ROS in cardiac cells. ROS stimulate NF-kB and pro IL-1b upregulation that progress the inflammatory pathways [30,31]. Abd-ElRaouf et al. (2021) reported that CYC induced cardiotoxicity via activation of the NF-κB signaling pathway [32]. In addition, Gunes et al. (2018) reported that CYC is a significant cause of renal toxicity [33].

In our study, the oxidative stress induced by CYC was manifested by increased MDA levels and decreased TAC in renal and myocardial tissues in contrast to the control group. These findings are consistent with studies demonstrating the ability of CYC to generate ROS, which contribute to lipid peroxidation and organ dysfunction [34,35,36]. Fortunately, TVF significantly decreased MDA levels while restoring TAC, at the higher dose, supporting its role in combating oxidative damage. The antioxidant effect of TVF may be attributed to its ability to stabilize cellular signaling pathways [37]. According to the study of Lawal et al. (2022), TVF–silver nanoparticles conjugate, TDF-AgNPs, prevented the death of prefrontal cortex (PFC) astrocytes and neurons by reducing cognitive deficits through the antioxidant qualities of silver nanoparticles [20].

The results of our study observed downregulation of Nrf2, HO-1, AMPK, Bcl-2, SIRT1, and LC3 in renal and myocardium tissues after CYC exposure highlights the impairment of cellular antioxidant, anti-apoptotic, and autophagic pathways. These findings align with previous works that reported that CYC effectively impaired antioxidant and anti-apoptotic pathways in many organs [38,39,40].

Nrf2 is a key transcription factor regulating antioxidant protection. Upon activation, Nrf2 translates into the nucleus and elevates the expression of HO-1, a critical enzyme for diminishing oxidative injury via degrading heme into biliverdin, free iron, and carbon monoxide. In our study, CYC-induced toxicity was shown to downregulate Nrf2 and HO-1, aligning with previous findings indicating oxidative stress as a regulatory mechanism of CYC-induced toxicity [41,42,43,44]. Hassanein et al. (2023) reported that upregulation of the Nrf2/HO-1 pathway exhibited an effective reno-protective effect [42], which was also reported by another study [45]. A study by Sherif et al. (2018) reported that CYC treatment revealed hepatic Nrf2 and HO-1 expression downregulation [46]. Matching with all these, our findings suggest that TFV may restore the cellular redox balance by activating the Nrf2/HO-1 pathway, consistent with its documented antioxidant properties [46].

AMPK is a cellular energy monitor that controls metabolic homeostasis [47]. Its activation is important for maintaining energy balance and inducing autophagy [48]. SIRT1 is an NAD+-dependent deacetylase with multifaceted function in oxidative damage resistance, inflammation attenuation, and regulation of the apoptotic pathway [49,50,51,52]. Recent studies highlight a significant protective interaction between SIRT-1 and AMPK to reduce CYC-inspired poisoning. SIRT-1 controls an NAD^+^-dependent deacetylase, cellular stress reactions, mitochondrial biogenase, and apoptosis. AMPK controls an energy sensor and metabolic homeostasis and is active under stress conditions. Both routes are paired, where SIRT-1 can activate AMPK through serine-threonine kinase 11 (STK11) deacetylation, and AMPK can increase SIRT-1 activity by increasing NAD^+^ levels [53].

In models of CYC-induced organ toxicity, co-activation of SIRT-1/AMPK has been shown to attenuate oxidative damage, reduce pro-inflammatory cytokines, and stimulate autophagy, thereby offering cytoprotection [53,54]. This axis is considered a promising target for protection against CYC-induced damage. Our results disclosed that CYC exposure led to significant AMPK downregulation, hindering energy homeostasis and increasing susceptibility to damage. Liu et al. (2016) reported that CYC induced renal toxicity via significant decrease in AMPK level and elevation in the inflammatory pathway [55]. The observed downregulation of SIRT1 in CYC-treated tissues is consistent with its role in facilitating apoptotic pathways and oxidative damage [56,57,58,59]. Additionally, Elrashidy et al. (2021) documented that CYC induced cardiotoxicity via downregulation of the SIRT1 signaling pathway [60].

Treatment by TFV reverses these effects via improving AMPK expression, aligning with its potential role in modulating metabolic pathways and improving cellular resilience [61]. AMPK activation can also enhance the Nrf2/HO-1 pathway, a critical antioxidant response pathway, as previously reported [62]. In particular, AMPK can stimulate the translation of Nrf2 to the nucleus, leading to elevated expression of protective genes such as HO-1 [62]. This in turn can reduce oxidative stress and inflammation and promote cellular repair mechanisms and boost the protective effect of TFV.

The ability of TFV to upregulate SIRT1 suggests the involvement of protective mechanisms, including enhanced mitochondrial function and anti-apoptotic signaling [22,23]. The upregulation of SIRT1 by TFV restores autophagic flux by enhancing LC3-II formation. This attenuates cellular damage and enhances tissue function [63]. Moreover, TFV’s ability to elevate SIRT1 expression leads to attenuated NF-κB activation and diminished levels of some pro-inflammatory cytokines, like IL-1β, that mitigate inflammation [64]. Additionally, restoration of SIRT1 via TFV-mediated activation of the Nrf2/HO-1 pathway improves antioxidant defenses and reduces oxidative damage [65].

Bax (pro-apoptotic) and BCL-2 family protein (anti-apoptotic) balance is essential for suppression of apoptotic pathway in tissues [66]. Our results reported that CYC injection resulted in disturbance of the balance between Bax and BCL-2, which revealed the significant reduction in BCL-2 levels in renal and cardiac tissues leading to elevated susceptibility to apoptosis as previously reported [67,68]. A study of El Kiki et al. (2020) reported that CYC induced cardiotoxicity and apoptosis via elevation of Bax and inhibition of BCL-2 protein expression [69]. Furthermore, Ijaz et al. (2022) reported that CYC induced nephrotoxicity via induced renal apoptosis [70]. Treatment with TFV remarkably attenuates this apoptotic pathway in both renal and cardiac tissues, and this is compatible with the anti-apoptotic properties of TFV.

Autophagy is a densely regulated cellular downturn required to maintain cellular homeostasis by removing damaged organelle and misfolded proteins. One of the most important markers of autophagy is proteins associated with microtubule 1A/1B-light chain 3 (LC3), which is widely used as an indicator for autophagy [71]. During starvation-induced autophagy, autophagosomes fuse with lysosomes, and the LC3 in autophagosomes is finally degraded by lysosomal proteases. Autophagy can be initiated in different stress conditions, but its completion (lysosomal degradation) can be impeded, resulting in LC3 accumulation. The dysfunction of lysosomes impedes the degradation of mitochondria, resulting in elevated production of ROS, consequently further impairing the lysosomes [71,72]. Autophagy disruption has been involved in various disease conditions, including cardiotoxicity and nephrotoxicity [73,74].

Several works have documented that CYC interferes with the autophagic process, either by elevated autophagy, which leads to cell death, or attenuating protective autophagic mechanisms. In our results, CYC-injected rats showed significant elevation in LC3 expression levels in kidney and heart tissues. Caglayan et al. (2018) reported that CYC injection elevated the expression of LC3, reflecting the distortion of the autophagic turnover, which contributes to liver and kidney toxicity [75]. In the heart tissue, CYC-induced stress has been changed to low autophagy activity and accumulated LC3 levels, which increases apoptosis and myocardial damage [58].

However, TFV contributed positively to the regulation of autophagy passage. TFV was found to restore autophagic flux, which was shown in the significant reduction in the expression level of LC3 in kidney and heart tissues. This restoration of autophagy contributes to the cytoprotective effects of TFV in both renal and cardiac tissues. Modulation of LC3 expressions by TFV matches its reported roles to maintain cellular integrity, reduce oxidative damage, and prevent apoptosis, and offers a multi-level protective system against CYC-induced poisoning [22].

### Limitations and Future Studies

One limitation of our study is that it did not investigate whether TFV affects the therapeutic efficacy of CYC or not. The second limitation is the absence of using pathway-specific inhibitors and gene silencing approaches to further delineate the mechanistic role of SIRT-1/AMPK/Nrf2 in mediating TFV’s protective effects. Our future studies will target the investigation of the possible impact of TFV on the therapeutic efficacy of CYC and the possible protective effect of TFV against other CYC-induced organ injury, such as liver and testes.

## 5. Conclusions

This study highlights that tenofovir (TFV) effectively reduces the toxic effects of cyclophosphamide (CYC) in rats by influencing key molecular processes related to oxidative stress, inflammation, apoptosis, and autophagy. When TFV was administered, it significantly lowered markers of kidney and heart damage, such as LDH, creatinine, BUN, and MDA. It also helped enhance the antioxidant defense system of the tissues by activating the Nrf2/HO-1 pathway and upregulating proteins like AMPK and SIRT1. Histopathological analysis confirmed the protective role of TFV, showing less damage and inflammation in the kidney and heart tissues. TFV also played a role in modulating apoptotic pathways; TFV increased anti-apoptotic Bcl-2 expression, contributing to cell protection, and lowered IL-1β levels. These findings suggest that TFV could be a valuable adjunct to help protect against the harmful effects of CYC, offering a promising strategy to minimize chemotherapy-induced organ damage. However, more research is needed to assess how TFV could be used clinically to enhance the safety of CYC treatments.

## Data Availability

The data of the current study are available from the corresponding author on request.

## Abbreviations

**Abbreviation**	**Full Name**
ACUC	Animal Care and Use Committee
AMPK	AMP-Activated Protein Kinase
BCL-2	B-Cell Lymphoma-2
BUN	Blood Urea Nitrogen
CMC	Carboxy Methyl Cellulose
CK-MB	Creatine Kinase-Myocardial Band
CYC	Cyclophosphamide
ELISA	Enzyme-Linked Immunosorbent Assay
HBV	Chronic Hepatitis B Virus
HIV	Human Immunodeficiency Virus
HO-1	Heme Oxygenase-1
ICAM-1	Intercellular Adhesion Molecule-1
IL-10	Interleukin-10
IL-1B	Interleukin-1B
IL-8	Interleukin-8
LC3	Microtubule-Associated Protein 1A/1B-Light Chain 3
LDH	Lactate Dehydrogenase
MDA	Malondialdehyde
NRF2	Nuclear Factor Erythroid 2-Related Factor 2
SEM	Standard Error of the Mean
SIRT-1	Silent Information Regulator Sirtuin 1
TAC	Total Antioxidant Capacity
TFV	Tenofovir
TNF-α	Tumor Necrosis Factor Alpha
TP	Total Protein
VCAM-1	Vascular Cell Adhesion Protein-1
